# Plural Causes

**DOI:** 10.1162/OPMI.a.345

**Published:** 2026-03-23

**Authors:** Can Konuk, Tadeg Quillien, Salvador Mascarenhas

**Affiliations:** Department of Philosophy, Stanford University, Stanford, CA, USA (work carried out at Institut Jean-Nicod, Ecole Normale Supérieure, Paris, France); Department of Psychology, University of Edinburgh, Edinburgh, UK; Department of Cognitive Studies, Institut Jean-Nicod, Ecole Normale Supérieure, Paris, France

**Keywords:** causal selection, counterfactual theories of causation, plurals

## Abstract

Causal selection is the process underlying our intuition that an outcome happened *because of* a given event, or that an event is *the cause* of an outcome. When a forest catches fire after a lightning strike, for example, people tend to say that the lightning bolt was the cause of the fire, not mentioning the presence of oxygen in the air, although they are well aware that the latter was no less indispensable for the fire to occur. The extant literature on causal selection has so far operated on the implicit premise that the only relevant variables for causal selection are *individual variables*, corresponding to distinct nodes in the relevant network of causes. Ours is the first systematic study of plural causes in the context of causal selection. First, we establish by means of two behavioral experiments the psychological reality and non-triviality of plural causes, ruling out potential deflationary explanations. Second, we show that state-of-the-art models of causal selection based on counterfactual dependence can be extended to make non-trivial predictions about plural causes consistent with our experimental findings. Third, we show that surprising logical properties of plurals *in natural language interpretation* might be found in causal reasoning with plural causes.

## INTRODUCTION

Causal selection is the process underlying our intuition that an outcome happened *because of* a given event, or that an event is *the cause* of an outcome (e.g., Hesslow, 1988; Quillien & Lucas, 2024). Causal-selection judgments go further than judgments of *actual causation* (Halpern, 2016; Halpern & Pearl, 2005; Hitchcock, 2001), whereby people merely identify which events can be counted as causes of an outcome. They induce a ranking over these events, singling out some as being more important than others in bringing about the outcome under consideration. When a forest catches fire after a lightning strike, for example, people tend to say that the lightning bolt was the cause of the fire, not mentioning the presence of oxygen in the air, although they are well aware that the latter was no less indispensable for the fire to occur. Causal selection in this sense is crucially distinct from *causal inference*, the problem of learning the relevant causal facts about the world. Causal selection concerns how we judge the relative importance of the many causes of an event, given that we already have a causal model of the situation.

A considerable literature has developed around what factors underly our preference for certain causal explanations of an outcome over others (Icard et al., 2017; Knobe & Fraser, 2008; Lombrozo, 2010; Morris et al., 2019; Quillien & Barlev, 2022). Although theories diverge as to what the drivers of causal-selection judgments are, they all agree that the outcome of causal-selection judgments depends crucially on the initial pool of candidates under consideration.

Before the lightning bolt can be viewed as *the cause* of the fire, the events *lightning*, *oxygen*, *dry season*, and others must first be flagged by the mind as relevant candidates for causal selection, whose relative importance in bringing about the outcome will be assessed. We argue here that the extant literature on causal selection has had a blind spot regarding that initial pool of candidates: it operates on the implicit premise that the only relevant variables for causal selection are *individual variables*, corresponding to distinct nodes in the relevant network of causes.

Instead, we argue that causal-selection judgments can recognize *plural* causes, featuring more than one variable, as when we say that “the dryness of the season and the strength of the wind” caused the spread of the fire. We argue that such plural causes are treated by the mind as candidate explanations on the same footing as the singular causes that compose them. In other words, people engage with such a conjunction of variables as if it were a coherent entity whose impact on the outcome should be evaluated in a wholesale fashion. And the same factors that drive the attractiveness (or lack thereof) of singular-cause explanations drive that of such multivariate causes. This contrasts with an alternative view in which causal selection is at its core merely about comparing the importance of “atomic” factors like *dry season* and *strong wind* that can in principle vary independently. On this view, the act of mentioning both in one’s explanation would merely be an acknowledgment that one does not have a preference between these two factors, or that each one is important enough that they deserve to be mentioned.

The idea that causal cognition admits causes featuring several variables is not in itself new. In causal inference, there is work on how people infer conjunctive causes, that is factors that act in concert to produce an effect (Novick & Cheng, 2004). The notion of a multivariate cause also plays a role in some theories of actual causation (e.g., Halpern, 2015), and, in a different way, in philosophers’ and economists’ concept of *collective responsibility* (e.g., Arendt, 1987; Miller, 2001). More specifically related to people’s choices of explanations, Lombrozo (2007) and Pacer and Lombrozo (2017) looked at participants’ preferences when given a choice between explanations that involved either one or several variables (see also Lucas et al., 2014, for related work with children). They observe that, everything else being equal, human adults prefer explanations that mention fewer unexplained variables (i.e., root causes with no causal parents in the graph). Such studies, however, concern judgments about the ground-truth causal system: which of several causal theories is most likely to be true. By contrast, causal selection, as it is understood here, is primarily concerned with situations in which the ground-truth causal theory—and with it, the variables that count as causes of an outcome—is presumed to be known already. In that context, it asks: which of these causes are most relevant to explain the outcome? To our knowledge, the literature on causal-selection judgments has yet to engage with plural explanations.

We present the first systematic study of plural causes in the context of causal selection.^1^ This study has three objectives. The first objective is to empirically establish the psychological reality and non-triviality of multivariate causes. In other words, that people assess the value of a plural explanation *A* ∧ *B* in a wholesale fashion, treating it as a candidate for causal selection on the same footing as the explanations *A* and *B* which mention events contained in it. To that effect we show two things. First, we show that people’s judgments about plural causes are sensitive to the prior probabilities of events, a key signature of causal-selection judgments. Second, and more importantly, we rule out a possible deflationary explanation for plural causes’ sensitivity to probabilities: that subjects might formulate a judgment about a plural cause like *A* ∧ *B* simply by combining in some direct way their judgments about the importance of the individual events *A* and *B* that compose it. In so doing we provide evidence that people treat plurals as full-fledged candidates for causal selection.

The second objective is to show how considering plural causes can expand our understanding of the role of counterfactual reasoning in causal judgments. We show that models of causal selection based on the notion of counterfactual dependence can straightforwardly be extended to make non-trivial predictions about plural causes consistent with most of our findings. Counterfactual models consider that the causal impact of an event *A* on an outcome *E* is a function of the extent to which *E* depends on *A* across counterfactual worlds sampled in a certain way. We show that, similarly, people’s intuitions as to the causal impact of a plural event *A* ∧ *B* is largely captured by the extent to which *E* depends on *A* ∧ *B* across counterfactuals. This cross-validates the counterfactual approach to causal selection by moving to a different class of judgments than the one it was originally developed for.

Our third objective is to explore how some factors other than mere counterfactual dependence as it is currently understood might play a role in subjects’ assessment of the contribution of plural causes. In our second experiment, we find surprising patterns in people’s judgments that cannot be explained by existing theories, particularly when explaining negative outcomes. We offer a tentative account of these findings, inspired by the formal semantics of natural-language plurals. This account speculates that the counterfactual simulation processes people use to evaluate the impact of causes might operate at the level of chunks of more than one variable. A computational model formalizing this proposal successfully captures the new unexpected results.

### Causation and Causal Selection

Humans are adept at representing the world through a web of causal relations between events. Representing causal relations allows people to make sense of what they observe, make predictions about what’s to come, and influence the future in some (Chater & Oaksford, 2013; Gerstenberg & Tenenbaum, 2017; Pearl & Mackenzie, 2018; Sloman & Lagnado, 2015).

In the psychological literature, people’s causal knowledge is usually modeled through formalisms such as Causal Bayes Nets or Structural Causal Models. These systems represent aspects of the world with variables, causal relations between these variables, and probability distributions (Pearl, 2000). They appear as integral parts of accounts of psychological faculties and functions related to causation, such as causal inference and counterfactual reasoning.

One such causation-related function is causal selection: faced with a complex causal structure, humans will gladly *select* one cause (or, as we will show, more than one) as being more important than others. Moreover, they will assign different scores to different causal variables depending on how they perceive each of those variables as being *the* driver of the observed outcome.

Knowledge of the causal rule in the relevant system is of course one of the main factors determining the explanation humans will favor in causal-selection judgments. The other main driver of causal selection is the *normality* attached to events, a notion that combines the extent to which an event abides by moral or conventional rules, and the extent to which it was expected to happen, before it did happen (Icard et al., 2017; Morris et al., 2019; Quillien & Lucas, 2024).

The relationship between the causal rule that entangles events with the outcome, their normality, and causal-selection judgments can be complex. In a situation where several different variables are each individually *necessary* for an outcome, people tend to think of the *least expected* variables (the lightning bolt) as *the cause*, and comparatively disregard the importance of the most expected variables (the presence of oxygen), a pattern of judgment known as *abnormal inflation*. The converse tendency is observed in situations where all of the variables considered are each individually *sufficient* for the outcome to occur. In this case, people tend instead to think of the most normal events as the most important causes of the outcome (Icard et al., 2017).

### Defining the Candidates for Causal Selection

Causal selection is determined by an amalgam of the system’s underlying causal rule and the normality of events. Thus, a standard procedure for formulating theories about participants’ causal-selection judgments starts by building a causal model that formalizes their causal knowledge of the system.

**Figure 1.**
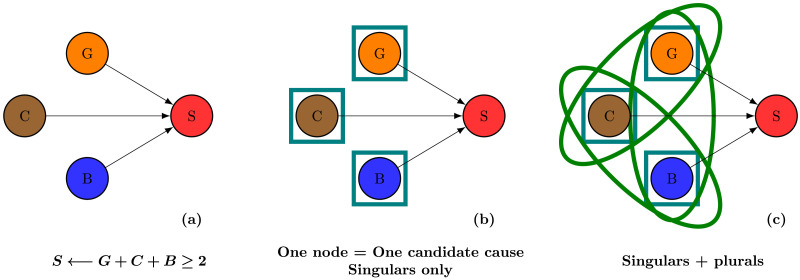
A causal model for the relations between various dishes and my stomachache. (**a**) I develop a stomachache if and only if I eat two pieces of pudding or more. (**b**) The standard implicit assumption in the literature is that only single variables are candidates for causal selection. (**c**) We propose instead that causal judgments can also target plurals, for example pairs of variables.

Suppose for example that I get a stomachache shortly after having eaten a piece of Gouda cheese and a plate of pudding containing chocolate cake and blueberry pie. A causal model of this situation would feature one variable for each of the causes of my stomachache (i.e., one variable each for “eating the Gouda cheese,” “eating the chocolate cake,” and “eating the blueberry pie”) as well as a variable for the effect (“having a stomachache”). The model also specifies a functional relationship between the variables, for example representing the fact that one develops stomach issues after eating too much, as schematized in Figure 1a.

As illustrated by this representational format, it is natural to think of the candidates for causal selection as particular realizations of the individual variables. If an individual equipped with the causal knowledge encapsulated by the model in Figure 1a wonders what *the cause* of their stomachache was, it may seem like they have to make a choice between the variables *G*, *C*, and *B*. This would directly identify the candidates for causal selection as the individual moving parts of the causal model, as represented in Figure 1b.

A striking feature of the psychological literature on causal selection is indeed that causal selection judgments are only ever queried at the level of singular variables (Kominsky et al., 2015; Morris et al., 2019; Quillien & Barlev, 2022; Quillien & Lucas, 2024; Sytsma, 2020). Kinney and Lombrozo (2024) deserve an honorable mention in this connection however, since they compared participants’ preferences for causal generics (“X causes Y”) mentioning one vs. several variables. But their work was on type causation, while here we discuss token (actual) causation.

Concretely, when experimental participants are presented with a situation where an outcome depends on three different events *A*, *B*, or *C*, they are never asked to what extent a *plural* event like *A* ∧ *B* can be considered the cause of the outcome. Intuitively though, causal explanations that mention combinations of variables can also be appealing. In our example above, saying that I got a stomachache “because I ate the entire dessert plate” might appear to be a better explanation than either “because I ate the chocolate cake” or “because I ate the blueberry pie” each on its own.

Note that allowing for many variables to feature in causal explanations does not eliminate the need for causal selection: one might want to mention several causes of an event without mentioning *all* of them. For example, one might think that “because I ate the blueberry pie” is a better explanation for my stomachache than “because I ate the entire dessert plate” if for example I eat chocolate cake at every meal, but add a blueberry pie on top of it only exceptionally. Ultimately, the best candidates for causal selection are those causes that participants see as most *crucial* in bringing about the outcome, whether these be singular or plural, and in principle we can only know what the best causal explanations are after considering the entire set of possible candidates, including plural causes, as illustrated in Figure 1c.

### Counterfactual Theories

To properly argue the point above, we first need to spell out what it means for a cause to be of a more or less crucial importance in bringing about an outcome. The notion we will rely on throughout this paper is rooted in counterfactual theories of causal selection (Icard et al., 2017; Quillien & Lucas, 2024).

Counterfactual theories of causal cognition in general build on the premise that humans represent causal relations between variables in terms of counterfactual dependence (Gerstenberg & Tenenbaum, 2017; Halpern & Pearl, 2005; Krasich et al., 2024; Lewis, 1973; Woodward, 2004, 2006). The notion that “*C* caused *E*” is taken to be roughly equivalent to the notion that “had *C* not happened, *E* would not have happened either.” In the case of causal selection judgments, this is enriched by evaluating counterfactual dependence not just once but across many possible worlds, asking how robustly *E* depends on *C* when background conditions vary. Of particular relevance to this evaluation will be the possible worlds that are most *normal*, or *closest to the actual world* in which we are to select a cause (Lewis, 1973). Evaluating counterfactual dependence in these worlds is what allows a causal-selection judgment to provide explanations that are not just relevant to the situation under consideration, but also generalize to other contexts (Hitchcock, 2012; Lombrozo, 2010).

We will limit our discussion in this article to two counterfactual theories (and accompanying models) of causal selection that (1) have been stated in full mathematical rigor and (2) have been submitted to experimental scrutiny, the Necessity and Sufficiency Model (Icard et al., 2017, NSM) and the Counterfactual Effect-Size Model (Quillien & Lucas, 2024, CESM). We chose to focus on these two theories because of their good track record in predicting participants’ causal selection judgments across a wide variety of tasks (Gerstenberg & Icard, 2020; Gill et al., 2022; Henne et al., 2019, 2021; Kirfel et al., 2021; Kominsky & Phillips, 2019; Morris et al., 2019; O’Neill et al., 2022, 2025; Quillien & Barlev, 2022).

The two theories see causal selection as a two-step process. The first step is identical across theories, the second divergent. The procedure is as follows.

**Figure 2.**
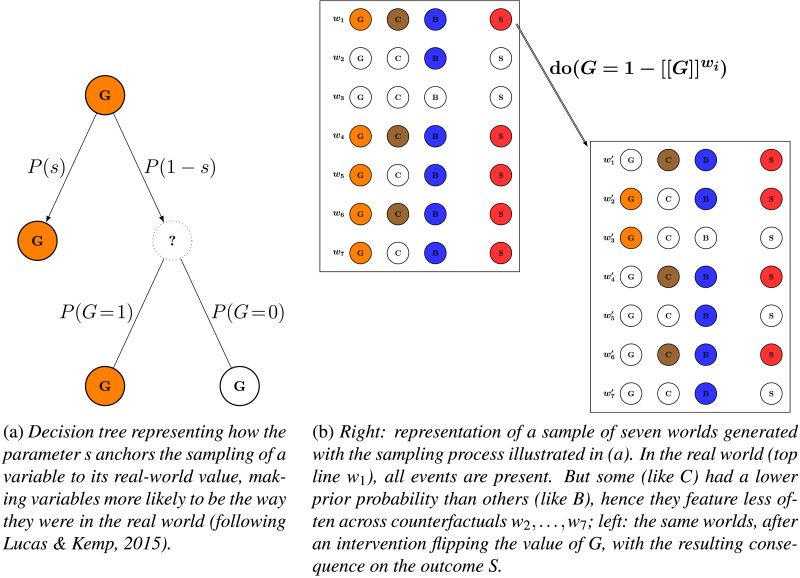
Sampling counterfactual worlds.

First, randomly sample a large number of counterfactual worlds. The sampling process operates at the level of the individual exogenous variables of the relevant causal model, that is, the variables that have no parent in the causal graph. In our stomachache example, these are the variables *G*, *C*, and *B* corresponding to the various dishes. To sample counterfactuals is to randomly decide, for each dish and in each counterfactual world, whether one eats that dish or not. The probability of doing so is determined by two factors:

1. *The value of the variable in the actual world*. With some probability *s*, corresponding to the *stability* parameter of the models, each variable retains the value it had in the actual world (Lucas & Kemp, 2015; Quillien & Lucas, 2024). For example, if I actually ate the blueberry pie, then in each counterfactual I simulate, there is at least probability *s* that I also eat the blueberry pie. This anchors counterfactual simulations to what actually happened, keeping them close to the actual world.
2. *The prior probability of the variable*. When a variable is not anchored to its actual values (which happens with probability 1 − *s*), it is resampled from its prior probability distribution. If I rarely eat blueberry pie (*P*(*B*) = 0.1) but frequently eat chocolate cake (*P*(*C*) = 0.8), then across counterfactuals, blueberry pie will be absent much more often than chocolate cake. This is how the models capture the influence of *normality*: abnormal (low-probability) events are more likely to be “switched off” in counterfactual simulations, making them more impactful if the outcome depends on their presence. Figure 2a illustrates this sampling process with a decision tree. Once the variables have been sampled in this way, the outcome in each counterfactual world is determined by applying the causal rule, as illustrated in Figure 2b.

Second, compute the causal impact of a given variable *V* across those counterfactual worlds. The two theories differ in how they measure this impact. According to the Counterfactual Effect Size Model (CESM), people make causal judgments by computing an effect-size measure that quantifies how much the value of variable *V* influences the value of variable *O*, across counterfactual possibilities (for formal details see Quillien, 2020; Quillien & Lucas, 2024). In the simple causal structures we study in this article, the model has a simple interpretation: the causal responsibility of *V* for *O* is the Pearson correlation between *V* and *O* across the counterfactual possibilities that people imagine.

The Necessity-Sufficiency Model (NSM) computes a weighted combination of two scores (see Icard et al., 2017, for formal details):

- *Necessity*: Starting from the actual world, we ask whether flipping the value of *V* would also flip the outcome. The necessity score is the probability that removing *V* removes *O*. In our example: given that you actually ate the chocolate cake and got a stomachache, would you have avoided the stomachache if you had skipped the cake?
- *Sufficiency*: Across counterfactual worlds where both *V* and *O* differ from their actual values, we ask whether restoring *V* to its actual value would also restore *O*. The sufficiency score is the probability that adding *V* produces *O*. In our example: in counterfactual scenarios where you neither ate the chocolate cake nor got a stomachache, would eating the cake have given you a stomachache?

The weighting between necessity and sufficiency depends on the prior probability of *V*. When *V* is rare (low prior), necessity dominates—we ask whether removing the rare event would have changed the outcome. When *V* is common (high prior), sufficiency dominates—we ask whether the common event reliably produces the outcome.

### Extending Causal Selection to Plurals

We hypothesize that humans consider plural causes as *bona fide* candidates for causal selection and that they assess their impact by tracking how an outcome counterfactually depends on them just like they would do for single-variable causes. The general idea can be explained simply: to assess the causal importance of a plural event *A* ∧ *B* is to look at the causal impact that this *compound event* has on the outcome of interest, using the same measures of causal impact that we summarized above for singular variables.

How, concretely, do we extend the CESM and NSM to evaluate plural causes? The key insight is that both models can be applied to any binary variable—and we can treat a conjunction like “A and B” as a single compound binary variable. This variable takes value 1 when both *A* = 1 and *B* = 1, and value 0 otherwise (cases where *A* = 1, *B* = 0, or *A* = 0, *B* = 1, or *A* = 0, *B* = 0). Once we define this compound variable, we can compute its causal impact using the same procedures described above.

For the CESM, we compute the correlation between this compound variable and the outcome across counterfactual worlds.

For the NSM, Necessity asks: would the outcome still occur if the cause had not occurred? When the cause is “A and B,” this means asking about counterfactuals where it is *not the case that both A and B occur*. This includes worlds where only *A* occurs, only *B* occurs, or neither occurs. The contribution of each counterfactual case is weighted by its sampling propensity.

We illustrate the computation of necessity in the context of our running example. For ease of exposition, we set *s* = 0, so that sampling propensities reduce to prior probabilities (see above for the role of *s* in anchoring counterfactuals to the actual world). Suppose we have eaten all three desserts and we got a stomachache (remember that one gets a stomachache if one eats two desserts or more). To compute the necessity score for the plural cause “chocolate cake and blueberry pie,” we fix the third variable “Gouda cheese” to the value it took in the actual world (in which cheese was indeed eaten) and then consider all ¬(cake ∧ pie) alternatives: cake-only (*P*(*C*) × *P*(¬*B*) = 0.8 × 0.9 = 0.72), pie-only (*P*(¬*C*) × *P*(*B*) = 0.2 × 0.1 = 0.02), and neither (*P*(¬*C*) × *P*(¬*B*) = 0.2 × 0.9 = 0.18) worlds. In the first two cases, two desserts are still eaten (one of the sweets, plus the Gouda cheese), so the stomachache still occurs and these cases contribute zero to the necessity score. Only the “neither” case flips the outcome. Since Necessity is a conditional probability, asking what fraction of cause-absent worlds would flip the outcome, we normalize by the total probability mass of these alternatives (0.72 + 0.02 + 0.18 = 0.92). Thus the necessity score is 0.18/0.92 ≈ 0.20.

Sufficiency asks: in worlds where the cause doesn’t occur and the outcome doesn’t occur, would the outcome occur if we made the cause occur? When the cause is “*A* and *B*,” this means asking about counterfactuals where we force both *A* = 1 and *B* = 1. We condition on worlds where the plural cause is absent (i.e., where it is not the case that both A and B occur) *and* the outcome is also absent. Sufficiency is then a probability-weighted sum: each conditioning world contributes 1 if forcing *A* = 1 and *B* = 1 would produce the outcome, and 0 otherwise; and this contribution is weighted by the world’s probability.

We illustrate with our running example. Suppose the probability of eating Gouda cheese is *P*(*G*) = 0.5. We must condition on worlds where the plural “chocolate cake and blueberry pie” is false and the stomachache is absent. The three conditioning cases and their probabilities are: cake-only without cheese (*P*(*C*) × *P*(¬*B*) × *P*(¬*G*) = 0.8 × 0.9 × 0.5 = 0.36), pie-only without cheese (*P*(¬*C*) × *P*(*B*) × *P*(¬*G*) = 0.2 × 0.1 × 0.5 = 0.01), and neither sweet, with or without cheese (*P*(¬*C*) × *P*(¬*B*) = 0.2 × 0.9 = 0.18). In each of these worlds, forcing both cake and pie guarantees at least two desserts, so each contributes 1, times its probability, to the sufficiency score. As with Necessity, we normalize by the total probability mass of the conditioning worlds (0.36 + 0.01 + 0.18 = 0.55). Since every conditioning world contributes its full weight, the sufficiency score of “chocolate cake and blueberry pie” is 0.55/0.55 = 1.

Finally, the overall causal score for a plural combines Necessity and Sufficiency, weighted by the prior probability of the plural itself. For “A and B,” this probability is *P*(*A*) × *P*(*B*). When the plural is rare (low *P*(*A* ∧ *B*)), necessity dominates; when it is common, sufficiency dominates. In our example, *P*(cake ∧ pie) = 0.8 × 0.1 = 0.08, so the overall score is (0.08 × 1) + (0.92 × 0.20) = 0.08 + 0.18 = 0.26. Note that, because the conjunction of two causes will always be less normal than each cause considered separately, the evaluation of a plural cause in the holistic version of the NSM will always involve putting more weight on the Necessity factor than one would for each cause considered separately.

This *holistic* account of plural causation within the CESM and NSM theories can be contrasted with a null hypothesis according to which people reconstruct their judgments about plural causes from their judgments about individual variables.

#### The Linear Combination Hypothesis.

An alternative view could indeed contend that people only ever have direct intuitions about the causal responsibility of the *individual* variables in their causal model, but can still make judgments about a plural cause say by adding up or averaging the individual causal strengths of its constituent variables.

For example, to compute how much they agree that “eating the chocolate cake and the blueberry pie caused the stomachache,” people might first compute their agreement with “eating the chocolate cake caused the stomachache” and “eating the blueberry pie caused the stomachache.” Then they might somehow aggregate the causal strength of each individual variable. We will call this the *linear combination* hypothesis. Under this hypothesis, each variable makes a fixed contribution to any plural it is part of, regardless of what other variables it is combined with: the contribution of “blueberry pie” to “blueberry pie & chocolate cake” would be the same as its contribution to “blueberry pie & cheesecake.”

This hypothesis is deflationary with respect to the psychological reality of plural causes in that it holds that people can make plural-cause judgments when prompted to do so, but they cobble them together from more primitive representations of causal strength at the level of individual variables. In such a view, the internal computations relevant to causal selection ultimately only attend to the individual components of the system. The sensitivity of plural causes to prior probabilities is merely a surface effect: one sees a plural *A* ∧ *B* as a better explanation than *B* ∧ *C* merely because it comprises a variable *A* with a better counterfactual dependence profile than the variable *C*.

### Overview of Experiments

In order to test our holistic account against the linear combination hypothesis, we need to identify contexts where the two accounts make different predictions about causal-selection judgments. This is what we do in our first experiment. This experiment establishes that plural causes are real psychological entities: to say that “*A* and *B*” caused an outcome is not merely to say that variables *A* and *B* are each individually relevant. It is to say that the outcome counterfactually depends on variables *A*, *B* taken together as a cluster, more so than on alternative candidates.

Having established this, in Experiment 2 we move on to explore how this clustering of variables interacts with the groupings implicit in the causal function that relate an outcome to all of its causes, such as when it accepts two possible sufficient conditions (*A* ∧ *B*) and (*C* ∧ *D*).

## EXPERIMENT 1

Our first experiment has the following goals.^2^ First, if plural causes are processed as genuine causes by the mind, factors that are known to affect causal-selection judgments should influence judgments about a plural cause. In particular, the probability of an event is known to affect judgments about whether that event caused an outcome (Morris et al., 2019). If plural causes are genuine candidates for causal selection, then we would expect analogous patterns to apply to them: varying the probability of events should affect causal judgments about whether a conjunction of these events is seen as causing the outcome. Second, we aim to rule out the deflationary *linear combination* account of the impact of probability on participants’ judgments. Evidence of non-linearity in causal judgments would constitute stronger evidence for the psychological reality of plural causes in human causal selection. We design a situation where both the CESM and the NSM predict that the causal strength of plural variables will not be a linear combination of the scores of individual variables. We compare their predictions to those of a null-hypothesis model that tries to predict the score of plural causes as a linear combination of the scores of individual variables.

### Methods

#### Design and Materials.

**Figure 3.**
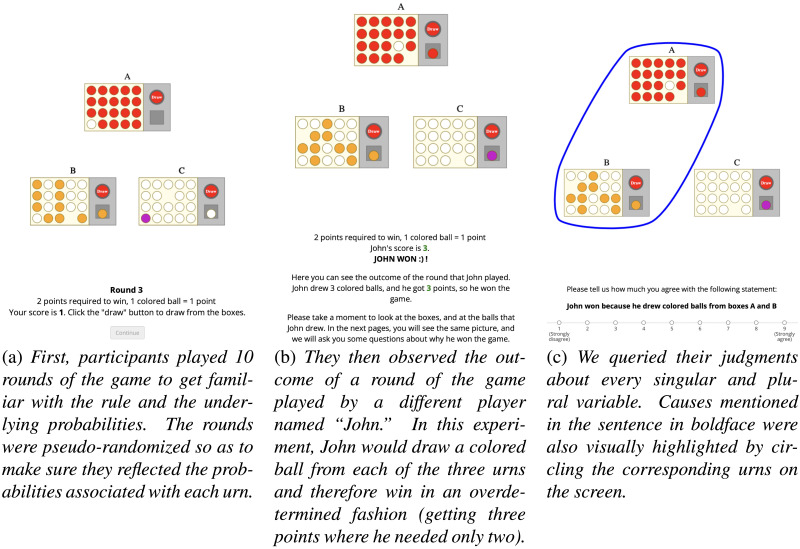
The three phases of Experiment 1.

We adapted a paradigm developed by Quillien and Lucas (2023). Participants made judgments about a game of chance, in which one randomly draws balls from a set of urns, and wins by getting enough colored balls (see Figure 3 for illustrations). Participants observed a fictitious player draw a colored ball from each of three urns (labeled *A*, *B*, and *C*) and win the game as a result. Then they were asked to make a causal judgment about each singular cause (e.g., whether getting a colored ball from urn *A* caused the player to win the game), and about each pair of causes (e.g., whether getting a colored ball from urns *A* and *B* caused the player to win the game). For exploratory purposes, we also asked participants to make a causal judgment about the triplet (getting a colored ball from *A*, *B*, and *C*). We manipulated the prior probability of each outcome within participants by varying the proportion of colored balls in each urn, with probabilities of 0.05, 0.5, and 0.95 (Figure 3). We will refer to the three different urns as the low, intermediate, and high urns, respectively. The rule of the game, which was directly revealed to the participant at the outset, was that the player wins if they get two colored balls or more. This corresponds to the causal model below.

WIN≔A+B+C≥2

#### Predictions.

This paradigm provides a context where the linear and the holistic extensions of the models we outlined above make clearly different predictions. The CESM predicts that participants’ singular causal-strength estimates should follow a particular ranking: intermediate > low > high, for any value of the *s* parameter. This is because, across possible counterfactual alternatives to what happened, there is a high correlation between getting a colored ball from the intermediate probability urn and winning the game. These predictions partially match participants’ judgments collected in the previous iteration of this paradigm run by Quillien and Lucas (2023), where judgments were collected for singular variables only, and in which participants’ responses followed the ranking: intermediate probability urn > low ≈ high. There was no significant difference between the ratings for the low probability and the high probability urn.

If we consider an extension of the CESM, linearly combining the predictions for individual causes, or alternatively if we simply combine participants’ judgments on individual variables, we would predict that participants should consider that the pair low & intermediate should have a causal strength greater than or equal to that of the pair high & intermediate, because the singular low has higher causal strength than high.

In contrast, if participants judge the causal strength of plurals via a holistic computation, they should rate the pair intermediate & high as highest. For across possible counterfactuals there is a high correlation between getting a colored ball from these two urns and winning the game. Intuitively, since drawing a colored ball from the low-probability urn is rare, and given that at least two balls are needed to win, most worlds where the player wins the game will be worlds in which they do so by getting a colored ball from the intermediate and high urns. This prediction is true for any value of the *s* parameter in the holistic version of the CESM. It is also true for the holistic version of the NSM, although in that case it reverses the ranking that the NSM expects for singulars.

#### Procedure.

Participants first completed ten rounds of the game, presented with urns as in Figure 3a. We pseudo-randomized the draws in such a way as to get participants to internalize the probabilities associated with each urn and how they connected to the outcome. Then participants saw the outcome of a round of the game played by another (fictitious) player, who drew a colored ball from all three urns, thereby winning with 3 points, as in Figure 3b. They were asked to rate the causal strength of each individual draw, as well as that of every combination of two or three draws for the winning outcome, on a Likert scale from 1 to 9 (strongly disagree to strongly agree), following standard practice in the causal-selection literature (Icard et al., 2017; Morris et al., 2019), as in Figure 3c. For the singulars, participants were asked to rate their agreement with the statement “John won because he drew a colored ball from box [urn].” For the plurals, they rated their agreement with “John won because he drew colored balls from boxes [urn1] and [urn2].” Each question was shown on a separate page, next to the urns that displayed the outcome of the fictitious player’s draw. The letters indexing the urns, as well as the colors of the balls, were randomized across participants but were kept the same for all trials within participants. Half of the participants were asked about the singulars first, and then about the pairs. The other half were asked about the pairs first, and then about the singulars. All participants were asked about the triplet at the very end. Within one class of questions (singulars vs. plurals) we randomized the order of presentation of questions. Finally, participants completed a brief demographic questionnaire and were redirected to Prolific for payment. We coded the experiment in the jsPsych library (De Leeuw, 2015), with custom plugins for displaying urns developed in our lab. A runnable copy of the experiment source code is available on the project's OSF page. A demo version of Experiment 1 can be accessed at https://konukcan.github.io/plural-causes-experiment-1/exp-1.html.

#### Participants.

We recruited 400 participants from all English-speaking countries from Prolific. This sample size was inspired by the one used by Quillien and Lucas (2023), who used a comparable sample size (290 participants) for a study with similar design. We excluded from subsequent analysis 41 participants who failed to answer either of two elementary comprehension questions and 3 whose data were incomplete, leaving a total of 356 participants for analysis.

#### Transparency and Openness.

We report how we determined our sample size, all data exclusions (if any), all manipulations, and all measures in the study. All data, analysis code, and research materials are available at https://osf.io/43m5d/. Data were analyzed using R (R Core Team, 2013) version 4.3.3 and the tidyverse package collection (Wickham et al., 2019), version 2.0.0. All studies we report in this article received ethics approval by the *Comité d’évaluation de l’éthique de l’INSERM*, under research protocol *Le langage et les capacités cognitives connexes*. All studies were conducted entirely in English. We did not preregister the studies.

### Results

We first report analyses using standard statistical tests. Then we report the fit of computational models of causal judgment.

#### Basic Results Patterns.

##### Prior Probability Affects Both Singular and Plural Causal Judgments.

**Figure 4.**
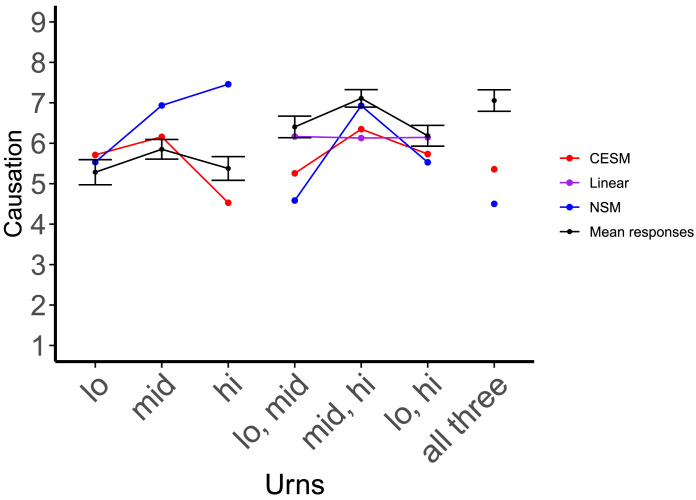
Mean ratings by question type, along with predictions for each theory under consideration. The error bars represent the standard error of the mean. The *linear combination* theory (purple) predicts that the score of the low & intermediate and intermediate & high pairs should be equivalent, when in fact we see a significant difference between them, in accordance with the *holistic* versions of the two counterfactual models: the Counterfactual Effect Size Model (Quillien & Lucas, 2024) (in red) and the Necessity and Sufficiency Model (Icard et al., 2017) (in blue).

Results are plotted in Figure 4. We ran two two-factor repeated-measure ANOVAs, one for each main type of cause queried (*singulars* and *pairs*), using urn probabilities and order of presentation as predictor variables, and participants’ responses as the dependent variable. Results are in Tables 1 and 2. There was a main effect of prior probability on participants’ causal judgments, for singular as well as for plural causes (*p* < 0.02 in both cases, see the tables for test statistics), consistent with our expectation that participants’ judgments of plural causes should be sensitive to probabilities just like for other actual-cause judgments.

**Table 1.** ANOVA for singular causal selection judgments, predicting urn ratings from urn probabilities and urn order of presentation.

Factors	Mean Sq	*F*-score	*p*-value
Probabilities of the urns	32.98	4.492	<0.012
Order of presentation	138.81	18.904	<0.0001
Probabilities:order	3.18	0.433	>0.64

**Table 2.** ANOVA for pair causal selection judgments.

Factors	Mean Sq	*F*-score	*p*-value
Probabilities of the urns	83.02	14.646	<0.000001
Order of presentation	21.75	3.837	>0.05
Probabilities:order	2.30	0.406	>0.66

The order of presentation/querying singular and plural selection judgments also had a significant effect (*p* < 0.001) on the ratings for singulars: singular causal judgments were lower when presented after the plurals. There was however no interaction effect between urn probability and order of presentation, suggesting that the impact of probability on causal estimates did not vary depending on the order in which questions were asked. Therefore we drop this variable (order of presentation) from later analyses.

##### The Causal Strength of Plural Causes Is Not a Linear Combination of the Causal Strength of Individual Variables.

The pattern of responses for singular variables replicated the patterns obtained by Quillien and Lucas (2023). Judgments for the intermediate urn were higher than judgments for the low urn, *t*(315.41) = −2.70, *p* = 0.007, and the high urn, *t*(325.85) = −2.08, *p* = 0.038. The difference between the low and high urns was not significant, *t*(350.59) = −0.63, *p* = 0.53.

We can use these results to test the *linear combination* hypothesis, according to which participants derive their plural-cause strength estimates by adding up or averaging their estimates for the individual variables that compose a given plural. If this were correct, participants should give the same causal strength estimate for the two plural causes low & intermediate and intermediate & high, since their estimates for the singular causes low and high are not significantly different from each other. By contrast, both the holistic CESM and the holistic NSM predict a sharp difference between these two kinds of plurals, with the plural cause intermediate & high being rated higher (Figure 4).

Consistent with the holistic CESM, judgments about the intermediate & high pair were higher than those for the low & intermediate pair, *t*(355) = −4.67, *p* < 0.001, and higher than for the low & high pair, *t*(355) = 6.858, *p* < 0.001. In a slight deviation from the CESM and NSM’s predictions however, judgments for the low & intermediate pair were higher than for the low & high pair, *t*(355) = 2.3691, *p* = 0.02 (Figure 4).

We conducted two more analyses to rule out the linear combination model. First, we ran a one-way repeated-measure ANOVA, predicting judgments for the pairs (low & intermediate, intermediate & high, and low & high) from judgments for the singulars (low, intermediate, and high), as well as their interactions, as within-participant factors. Each plural pair was regressed only on the values of the two singulars that comprised it.

The linear combination theory predicts that there should be no significant interaction: a participant’s causal judgment for a given *singular* variable should have the same impact on every plural cause in which it features. One’s estimate for the singular intermediate, for example, should have an equal impact on one’s estimate for intermediate & high and for low & intermediate.

We find evidence against the linear combination theory (Table 3). There was a significant interaction between the intermediate and high urns, *p* = 0.004. In addition, the main effects of the singular judgments were not significant, for all but the low urn.

**Table 3.** Results of the ANOVA: estimate for pairs ∼ est. for singular-1 × est. for singular-2.

Factors	Mean Sq	*F*-value	*p*-value
low	100.69	17.743	<0.00001
intermediate	20.80	3.664	0.05586
high	15.93	2.808	0.094
low:intermediate	6.64	1.169	0.2798
low:high	0.41	0.073	0.7872
intermediate:high	46.64	8.219	0.00423

Second, we fitted linear multilevel regression models on participants’ responses for pairs. Specifically, we compared the predictive performance of two different models on participants’ plural-cause estimates. The first one used as predictor the average of the two singular-cause estimates for the variables contained in a given plural (computed on a per-participant basis), plus a random intercept. The second model also included the question asked (that is, the specific plural being queried) as predictor. A likelihood-ratio test shows that adding question as a predictor significantly improves the fit of the model, *χ*^2^(5) = 27.53, *p* < 0.001 (Table 4). Again, this is inconsistent with the linear-combination account.

**Table 4.** Comparison between two models: the linear combination model of plurals (means of singulars + intercept), and the means of singulars + question model.

Models	LogLik	Df	*χ* ^2^	*p*-value	BIC
Means sing	−891.89	3			1804.709
+ Question	−878.13	5	27.53	<0.00001	1791.126

#### Computational Modeling.

We computed the predictions of two recent counterfactual models of causal selection, the Counterfactual Effect Size Model (Quillien & Lucas, 2024) and the Necessity and Sufficiency Model (Icard et al., 2017), presented in the introduction. Our implementation follows the one given by Quillien and Lucas (2023).

For each question we report on below, we generated causal judgments for the CESM using a process of counterfactual sampling. We generated predictions for the CESM by simulating 10^5^ possible rounds of the game according to the rule, what was the case in the situation described to participants, and the sampling model described by the CESM. We computed CESM judgments for an event as the correlation between that event (for instance, whether the player draws a colored ball from urn *A*) and the outcome of the game (whether the player wins the game), across simulations. We computed NSM judgments analytically, as the sum of the variables’ sufficiency and necessity scores across worlds.

We fit the value of the stability parameter *s* for both models by finding the value of *s* that results in the best fit between model judgments and average participant judgments across all seven questions. We quantified model fit by looking at the likelihood of mean answers per question under a normal distribution centered on the model’s predictions, with a standard deviation fitted across questions.

We identified the best fit value via a grid search, exploring a wide range of values for the parameter *s*, crossed with different values for a scaling parameter *γ* (applied to a model’s predictions as an exponent *prediction^γ^*). The point of *γ* was to avoid situations where one model would systematically overshoot or undershoot actual participant answers, as the models are not meant to predict the exact value of participants’ judgments, but only the relative difference between one variable and the next. Our technique here was analogous to that of Griffiths and Tenenbaum (2005).

For the CESM, the best fitting value was *s* = 0.89, with *γ* = 0.26. For the NSM, the best fitting value was *s* = 0.71, with *γ* = 2.93.

In our implementation, to assess the causal strength of plural causes a model assumes that people compute the causal strength of the conjunction of all variables contained within that plural. For instance, the CESM computes the causal strength of low & high by computing the correlation between the compound binary variable low ∧ high (which has value 1 if both low and high have value 1, and 0 otherwise) and the outcome.

The predictions of the models are plotted in Figure 4. Table 5 details the comparison. Overall, the CESM’s predictions had the best fit to human judgments in this experiment, although the NSM had the best fit when models were compared on pairs of variables only. We also compared the models’ performance on the judgments for pairs to a null model that used as predictor for each pair the average of mean human judgments for each singular variable contained within a given pair, as plotted in Figure 4. Both counterfactual models proved significantly better than this linear predictor (Table 5).

**Table 5.** Model comparison for Experiment 1, excluding the triple. The AIC and BIC values are computed for mixed effects models, including group and a random effect for participants.

Model	AIC	BIC	Cor.
CESM	9929.31	9957.65	0.54
NSM	9962.06	9990.4	0.24
Considering only the pairs			
CESM	4692.11	4716.978	0.83
NSM	4674.355	4699.222	0.80
Empirical average	4692.942	4717.81	−0.65

### Discussion

We find evidence that, when people make a judgment about whether events *A* and *B* caused an outcome, their judgments track the correlation between the conjunction of *A* and *B* and the outcome, across counterfactuals. Concretely, in our experiment, winning the game is in general strongly associated with getting a ball from both the intermediate and high-probability urns, and people judged that combination of events to be highly causal. Importantly, this effect is inconsistent with a simpler account, according to which people’s judgments about plurals are cobbled together from their causal intuitions about each individual variable in the plural.

Judgments about plural causes are affected by the prior probability of their constituent variables, but cannot be derived from the causal strength of these individual variables. As such, our results are in general consistent with the predictions of simple extensions of recent counterfactual models of causal selection (Icard et al., 2017; Quillien, 2020; Quillien & Lucas, 2024), augmented with the assumption that people judge plural causes in a holistic manner.

At the same time, these findings raise new questions about the psychology of causation. Presently we highlight two of these questions, which we investigate in Experiment 2.

First, participants in this study found the plurals overall more appealing than the singulars, a tendency which the counterfactual models we considered did not capture. Participants might have felt that plurals provided more exhaustive descriptions of the event: they give more complete information about what happened, in addition to why it happened. We also find that this effect is accentuated when singulars are presented after plurals. Making judgments about plurals first might highlight to participants the descriptive incompleteness of singulars. This finding suggests an interesting tension between two potential desiderata of causal judgment: highlighting the variables that were most causally important to the outcome, and providing an exhaustive list of the causal factors. If so, this calls for an investigation into the relative importance of these two pressures in participants’ causal-selection judgments. When, for example, adding a variable weakens the counterfactual dependence profile of the resulting plural, such as when the plural doesn’t explain the outcome appreciably better than one of the singular variables within it, will participants still show a preference for plurals, on account of their greater completeness?

Second, a notable feature of this first experiment is that the causal structure used a simple additive rule (i.e., the player wins the game if their score is above a certain threshold). As such, there is a sense in which the variables each have an independent incremental causal effect on the outcome.

What will participants’ plural-cause judgments look like in a causal structure where some conjunctions of events directly feature as such in the causal model that generates the outcome? Consider for example the causal rule (*A* ∧ *B*) ∨ *C*. Here the urns *A* and *B* are specifically connected in the logical structure. Generalizing somewhat, our question here is: when an outcome specifically depends on the joint occurrence of *A* and *B*, should that make the plural cause *A* ∧ *B* a more natural causal explanation than a potential alternative *A* ∧ *C*, even if *C* also makes an important contribution to the outcome?

## EXPERIMENT 2

Experiment 1 established the psychological reality and relevance of plural causes for causal-selection judgments. It provides evidence that, in order to assess the value of a conjunctive explanation like “*E* happened because of *A* and *B*,” people would track the way in which the group constituted by variables *A, B* contributes to *E* across counterfactuals. Building on these findings, Experiment 2 expands our exploration of plural causes by looking at a richer causal structure. Here, two urns contain purple balls, and two urns contain orange balls. The player can win the game by getting either two purple balls or two orange balls, where “or” is meant inclusively. Formally, winning can be triggered by either of two distinct sufficient conditions *A* ∧ *B* and *C* ∧ *D*, each a conjunction of two variables. This corresponds to the rule

$$\text{WIN}≔\left(A\wedge B\right)\vee \left(C\wedge D\right).$$ (1)

The point of moving to such a richer rule is fourfold.

First, we provide additional evidence against deflationary interpretations of plural causes. We give more examples of situations in which people’s plural-cause judgments cannot be straightforwardly derived from a linear combination of their singular-cause judgments, to confirm the results obtained in the first experiment.

Second, we explore whether there is a robust bias toward preferring causes that contain more variables. In Experiment 1, participants gave overall stronger scores to plural causes than singular ones. Experiment 2 investigates whether this pattern always holds.

Third, we study how the groupings involved in plural explanations interact with those implicit in the causal rule linking variables to their outcome. Intuitively, given knowledge of the rule (1), and of the value of variables *A, B, C, D* in some (actual or counterfactual) world, in order to assess that a round is won one might want to check each sufficient condition separately: whether *A* ∧ *B* is true, and whether *C* ∧ *D* is true. The two mental operations might run in parallel; but what matters is that each pair is attended to separately, therefore inducing its own implicit grouping, which in this case follows the boundaries of the logical disjunction.

This raises the question of how such implicit groupings will affect the way in which subjects like to group variables when they engage in plural explanations. Counterfactual models per se are not committed to the idea that such groupings matter for the evaluation of causes. They only attend to counterfactual dependence, which may favor a conjunction of variables borrowing from both disjuncts, like *A* ∧ *C* over one that maps onto just one, like *A* ∧ *B*. Both of the counterfactual models we considered indeed make such predictions in the context of this experiment. An interesting possibility to explore is that people might be evaluating the contribution of causes by looking at how much they contribute to one of these two paths, rather than directly looking at how they correlate with the outcome itself. To foreshadow our results, we did find some evidence in favor of such a view, by showing that people tend to dislike plural explanations that “cross” the disjunction, in a way that goes above and beyond what is predicted by the counterfactual dependence profile of these variables alone.

Fourth, we explore participants’ judgments in situations where they have to explain a *negative* outcome. In the context of our experiment, this amounts to explaining a loss. We call losing a *negative* outcome here, not in the sense of an undesirable outcome, but in the sense that subjects have only been positively instructed in the sufficient conditions for winning. The conditions for losing are merely contained *implicitly* in these instructions, as the negation of winning conditions. As we detail presently, this contrast opens interesting possibilities about the representations participants might have for losing conditions.

Finally, Experiment 2 was also designed to collect many more data points per participant, increasing our statistical power compared to Experiment 1. We ask each participant about the outcome of four possible rounds of the game (as opposed to just one outcome in Experiment 1), collecting a total of 36 causal judgments per participant.

### Negative Outcomes and Plurals

Responsibility attributions for negative outcomes are relatively understudied. We included losing rounds in Experiment 2 for completeness and for exploratory purposes, looking to see how the patterns of judgments we would observe for wins would transfer to losing outcomes. As we will see shortly, the results turned out more surprising than we anticipated, so it is worth foreshadowing these results with some discussion of what one might *a priori* expect the relationship between winning and losing outcomes to be.

Part of the reason why negative outcomes are understudied might indeed come from the expectation that the shift from positive to negative should be trivial, especially for binary variables. The same processes by which we assign responsibility to wins can be repurposed for losses, simply by moving the target. Erstwhile “wins” now count as losses and “losses” become wins, as far as assigning credit to causes goes. This would be true if we assume that the processes by which counterfactual outcomes are determined amount to consulting a model that already matches each assignment of values to causes to an outcome. If the model can tell which configurations of urn draws are winning, then it can *de facto* also tell which ones are losing, as those are just the complement of the wins. In which case, explaining a loss would simply amount to tracking how different causes co-vary with the *classical logic negation* of winning conditions across counterfactuals.

But available evidence suggests a more complex picture. Recently, in a study of *ex ante* responsibility judgments (that is, judging the importance of various causes before any outcome has effectively occurred or the value of any causal variable is known), Gerstenberg et al. (2023) observe that subjects’ estimates are better captured by a measure that tracks a variable’s contribution to wins than one that tracks its contribution to losses (or some hybrid of the two). This suggests that positive outcomes are taken to be the explananda by default for responsibility judgments. Knowing what it takes to win, one may go about checking whether these conditions are satisfied in each counterfactual, and credit causes as a function of how they contribute to satisfying these conditions.

But when people try to explain a loss, they might not simply reconstruct the losing conditions by computing the classical logical negation of winning conditions, and then check if and when these losing conditions are satisfied. A case in point here is provided by a series of experiments by Gerstenberg and Icard (2020), who looked at causal-selection judgments in a billiard-ball setting involving simple conjunctive or disjunctive rules. The researchers collected subjects’ estimates in positive cases where two events *A* and *B* happened, and in negative cases where both *A* and *B* failed to happen. As they noted, judgments for negative outcomes in the *disjunctive* cases where *E* ≔ *A* ∨ *B* were neatly captured by treating negative outcomes as the classical negation ¬*E* ≔ ¬*A* ∧ ¬*B*. But the same strategy did not work in the conjunctive case where *E* ≔ *A* ∧ *B*.

The results in our Experiment 2, whose causal rule involves a combination of conjunction and disjunction, provided another striking example. Subjects’ judgments for losing rounds diverged sharply from what one would expect if losses were scored by the same processes as wins, just applied to the negated rule. As a result, counterfactual models tracking how different causes co-vary with the *classical logic negation* of winning conditions did not do a very good job at capturing the data. These are striking empirical findings, relevant to anyone working on causal selection. They challenge not just specific counterfactual models, but the broader “moving the target” assumption: that whatever dynamics govern causal selection for positive outcomes should carry over to negative ones.

This raises the question of what sort of new dynamics come into play when people engage with negative outcomes in the context of causal selection. Raising this question is, we believe, one of the principal contributions of the present study—one that stands on its own, regardless of how one ultimately answers it. We remain open to different accounts, but offer one possibility that we find illuminating: the issue may lie not in the counterfactual machinery itself, but in what people take to be the target when scoring causes for negative outcomes. Counterfactual models work by matching causes against outcomes across simulated possibilities. For wins, the target is clear: the winning conditions. For losses, one might assume the target is simply the classical negation of those conditions—but this is not the only option. One possibility we explore is that people score the contribution of causes to negative outcomes by matching them against a representation of losing conditions that departs from classical logic, in a way inspired by how natural language handles the negation of plurals. Crucially, our stimuli contain no negation words—participants read about “losing,” not about “not winning.” So whatever role negation plays here, it must be in how people internally construct the target they score causes against, rather than in the literal phrasing of the question.

As we will show, the patterns we observe turn out to be consistent with the hypothesis that participants represent losing conditions using the non-standard formula

LOSS≔¬A∧¬B∧¬C∧¬D.

Note that this is not the correct equation one would obtain by applying classical logical negation to the equation for winning the game. Nonetheless, a counterfactual model that assumes that people simulate counterfactual possibilities in this *non-classical* way provides a very good fit to the judgments collected in the losing rounds of our experiment. Of course this finding raises the question: why would subjects rate explanations as if they endorsed a representation of the losing conditions that is incorrect? And also: why (as suggested by the data from Gerstenberg & Icard, 2020) would they only engage in such non-classical scoring procedures when dealing with conjunctions, but not with disjunctions?

One possible interpretation is suggested by the phenomenon of *homogeneity* observed for plurals in natural language. Plural entities in natural language have the logically surprising feature that negation applies homogeneously to each individual in the plurality. Consider the examples of plurals in (1) and (2), and the putative interpretations for the negated plural (2) in (2a) and (2b).

(1)  The boys did their homework.
(2)  The boys didn’t do their homework.
  (a)  None of the boys did his homework.
  (b)  At least one of the boys didn’t do his homework.

Sentence (1) means that *every* boy did his homework, with some tolerance for exceptions which needn’t concern us here (Križ & Spector, 2021). Sentence (2) then ought to be simply the negation of (1), which would amount to the interpretation paraphrased in (2b). Yet, the negated plural in (2) has a much stronger interpretation, to the effect paraphrased in (2a). In general, negated plurals are interpreted in this unexpected way, from the standpoint of classical logic (Krifka, 1996; Lappin, 1989; Löbner, 2000).This observation applies to plurals as in (2), generated by a noun phrase with plural morphology “the boys,“ but also to plurals formed by means of an explicit conjunction: a sentence like “John and Mary don’t speak German“ means that neither John nor Mary speak German, not merely that at least one of John or Mary doesn’t speak German (but see Szabolcsi & Haddican, 2004, for evidence of cross-linguistic variation on the available interpretations).

In light of these observations, one possibility worth exploring is that participants in our experiment score candidate explanations for losses by tracking the contributions of candidate causes to the stronger losing conditions obtained by negating the sufficient winning conditions in Equation 1, which is a disjunction of plural terms, in this non-standard way. This would amount to the stronger loss conditions at the end of Equation 2 below, which we preface with ‘≡’ to indicate that it violates classical-logical equivalence.

LOSS≔¬A∧B∧C∧D≡¬A∧B∧¬C∧D≡¬A∧¬B∧¬C∧¬D=LOSSstrong (2)

To be very clear, we are *not* saying that we expect people will *misinterpret* the rules of the game. We do not expect that they would classify, say, a round of the game where the player only draws colored balls from urns *A* and *C* as anything other than a loss, any more than we expect English speakers that take (2a) to be the natural interpretation for (2) would mistake (1) for a true sentence, had exactly one boy done his homework. Instead, our hypothesis is that the tendency to negate plurals in this homogeneous way shapes the model that participants use as target to match for computing the contribution of various causes to losses across counterfactuals. Just like people assess a cause’s contribution to wins by matching it against sufficient conditions {*A* ∧ *B*, *C* ∧ *D*}, they score its contribution to losses by matching it against the plural negation of those, which yields LOSS*_strong_*.

### Methods

#### Design and Materials.

**Figure 5.**
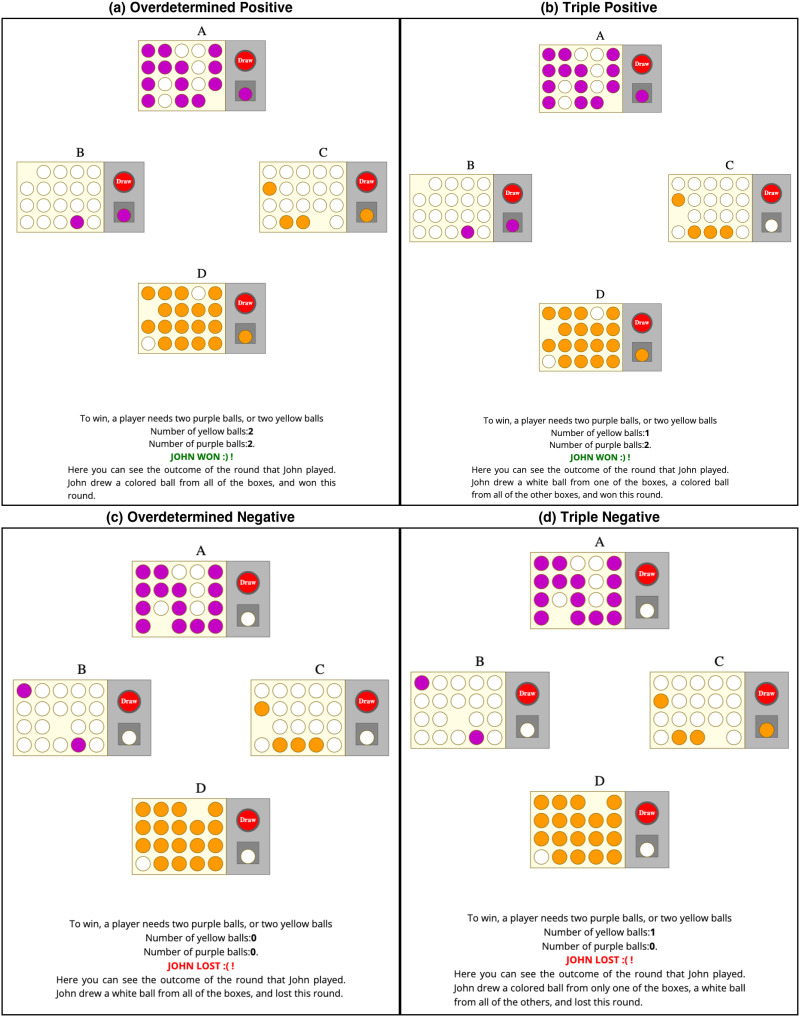
The four different outcomes presented to participants in Experiment 2. The familiarization phase, where people would get used to the underlying rule of the game and to the probabilities, was the same for all rounds. The questions were asked in a format analogous to that of Experiment 1.

The methodology was similar to that of Experiment 1. We presented participants with a simple game of chance. This time, the game involved four urns, with two different colors, purple and yellow (Figure 5). We randomized the assignment of colors, but always in such a way that urns *A* and *B* were of one color, and urns *C* and *D* of the other color. To win a round of the game, one needed to draw “two purple balls or two yellow balls.”

While we randomized the specific urns’ indices and their spatial arrangement for each participant, for simplicity here we refer to a consistent arrangement as depicted in Figure 5, where urn *A* has 14 colored balls, urn *B* 2, urn *C* 4, and urn *D* 19 colored balls. These induce different prior probabilities of drawing a colored ball out of each urn, such that *P*(*A*) = 0.7, *P*(*B*) = 0.1, *P*(*C*) = 0.2, and *P*(*D*) = 0.9. Throughout the experiment, the urn containing 14 colored balls and the urn containing 2 colored balls were always of the same color, while the other two urns (19 and 4 colored balls) were of the other color, so that each color would contain one high probability and one low-probability urn.

#### Procedure and Participants.

As in Experiment 1, participants first had the opportunity to familiarize themselves with the game and the rule determining a winning outcome, as well as with the underlying probabilities, by playing the game for ten rounds, as in the first Experiment. Urn draws and outcomes at this stage were pseudo-randomized in such a way as to reflect the underlying probabilities. The experiment source code is available on the project's OSF page. A demo version of Experiment 2 can be accessed at https://konukcan.github.io/plural-causes-experiment-2/exp-2.html.

After they played ten rounds of the game, they saw the outcomes of rounds played by another player named John (as in Figure 5) and were asked to rate on a Likert scale from 1 to 9 the causal responsibility of certain events, both singular and plural. Specifically, we queried their causal judgments by asking them the extent to which they agreed (on a 1–9 scale) with a sentence that followed the template: “John won (/lost) because he drew colored (/white) balls from box(es) [XYZ].” The questions were asked in a format analogous to that of Experiment 1.

All participants saw four different rounds of the game played by John, one at a time, and provided their judgments after each round. All the rounds were played with the same underlying rule and the same urns in the same display as the one participants had been familiarized with. Each trial differed only in the outcome of the draw made by John. We presented all participants with the following four rounds, in random order:

1. overdetermined positive: John drew a colored ball from each of the four urns—John won (Figure 5a)
2. triple-positive: John drew a colored ball from urns *A*, *B*, and *D*, but not from urn *C*—John won (Figure 5b)
3. triple-negative: John drew a white ball from urns *A*, *B*, and *D*, but not from urn *C*—John lost (Figure 5e)
4. overdetermined negative: John drew a white ball from all four urns—John lost (Figure 5c).

Within each round, we asked participants about every singular event that featured a colored ball in the winning rounds, and every singular event that featured a white ball in the losing rounds. We also asked about every plural combination of these singulars, with the exception of four-variable plurals (we considered those questionable candidates for causal selection judgments, since they provided an exhaustive description of all drawing events in a given round) and other plurals which we considered redundant with some that we already asked. The questions were presented in random order, with no separation between singulars and plurals.

We recruited a total of 368 participants (153 male, 215 female, mean age: 37.3) from all English-speaking countries on Prolific. We excluded from analysis 57 participants who failed to correctly answer either one of our two elementary comprehension questions, yielding a final sample of 311 participants whose data we analyzed. Each participant answered all of the questions of the four conditions in this experiment.

#### Computational Modeling.

We computed the predictions of the CESM and the NSM following the same procedure as in Experiment 1. We fitted the value of *s* and *γ* for both models by finding the parameter values that resulted in the best fit between model judgments and average participant judgments across all four conditions. As in Experiment 1, we used a grid search, exploring a wide range of values for the parameter *s*, crossed with different values for a scaling parameter *γ*. For the CESM, the best-fitting value was *s* = 0.21 (with *γ* = 0.39). For the NSM we find *s* = 0.02 (with *γ* = 0.28).

We also explored a variant of the computational models that allows for the possibility that participants handle the losing cases in the non-classical fashion discussed above. We provide the details of this model in the relevant subsection of the results.

To build intuition for how these models apply to Experiment 2’s richer structure, it helps to work through a concrete example. The same mechanics from the simpler contexts in the Introduction and Experiment 1 carry over here, just applied to a more complex causal rule. Consider the overdetermined positive round, where *A* = *B* = *C* = *D* = 1: the player drew colored balls from all four urns and won. As in Experiment 1, counterfactual worlds are sampled using the same process, governed by the same parameters.

Take the cross-disjunct plural “*A* and *C*.” For the CESM, we ask how the presence or absence of *A* ∧ *C* correlates with the outcome. In worlds where *A* ∧ *C* is true, the outcome is almost always positive: *C* is present and *D*, being high-probability, is usually present too, so *C* ∧ *D* is satisfied. As for worlds where *A* ∧ *C* is false, most are worlds where *A* is present but *C* is absent. In these worlds, the outcome is usually negative: *C* ∧ *D* fails because *C* is absent, and *A* ∧ *B* usually fails because *B* is often absent. In short, the truth value of *A* ∧ *C* tracks the outcome closely, yielding a high CESM score.

For the NSM, recall that Necessity measures how often removing the cause flips the outcome. For “*A* and *C*,” this requires counterfactual worlds where both *A* = 0 and *C* = 0: only then do both disjuncts fail, flipping the outcome to negative. This is a relatively small fraction of worlds as again, most worlds where *A* ∧ *C* is false are worlds where the high-probability variable *A* is present but the low-probability *C* is absent. But it is non-zero, which puts it at an advantage against same-disjunct plurals such as “*A* and *B*,” whose negation can’t flip the outcome in the present context, because *C* ∧ *D* would still be present. Sufficiency, on the other hand, is high: in worlds where *A* ∧ *C* is absent and the outcome is negative, forcing *A* = 1 and *C* = 1 almost always produces a win, because *D* is usually present, so *C* ∧ *D* is satisfied. The overall NSM score combines Necessity and Sufficiency weighted by the prior probability of the cause. For “*A* and *C*,” with prior *P*(*A*) × *P*(*C*) ≈ 0.14, most weight falls on Necessity. Since Necessity is non-zero but modest, the combination gives “*A* and *C*” a decent NSM score, higher than most same-disjunct pairs.

### Results

We first go through the results for each round separately. We start each section by a brief exposition of the predictive performance of the CESM and NSM models for the round, before delving into a qualitative analysis of the relevant patterns of judgments observed for that round. Note that none of the patterns we identify or the interpretation we provide for them depend on the models considered, unless explicitly specified otherwise. We provide these predictions mainly for readers interested in how state-of-the-art counterfactual models fare at predicting these new data.

#### Winning Rounds.

##### Overdetermined Positive Round.

In this round, the player drew a colored ball from each of the four urns (as in Figure 5a) and therefore won the game.

**Figure 6.**
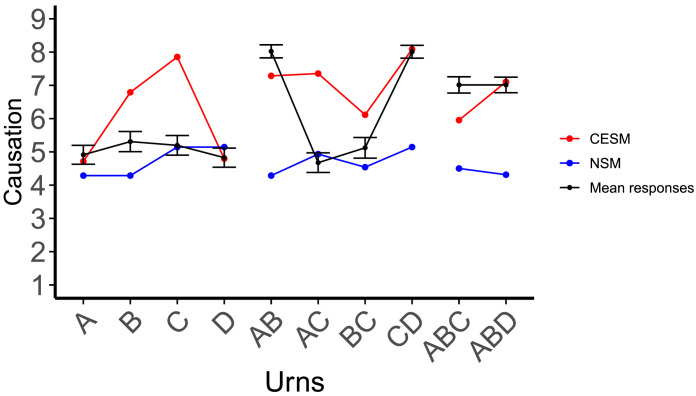
Participants’ responses, along with model predictions, for the overdetermined positive round. The red line represents the CESM predictions, the blue line the NSM predictions, and the black line represents the mean of participants’ responses.

Figure 6 summarizes our results in this condition. The CESM had a moderate but positive fit to participants’ average judgments, *r*(8) = 0.45, while the NSM predictions were uncorrelated with participants’ judgments, *r*(8) = −0.18.

Participants’ judgments also reveal the following patterns.

#### Non-Linearity.

Participants judged that *B* and *C* were the most important singular causes. Therefore, a linear combination approach would predict that they should also view the pair *B* ∧ *C* as the best plural cause. In fact, participants judged that the pairs *A* ∧ *B* and *C* ∧ *D* were significantly better causes than *B* ∧ *C*, in clear opposition to the predictions of the linear combination hypothesis.

#### Participants Preferred Non-Crossing Over Crossing Pairs.

There was a clear preference for pairs that did not cross the disjunction (*A* ∧ *B, C* ∧ *D*) over those that featured one variable on each side of the disjunction (e.g., *A* ∧ *C, B* ∧ *C*), (mean non-crossing: 7.02, mean crossing: 4.90; *t*(1055.63) = 23.97, *p* < 0.0001).

#### Weak Abnormal Inflation for Singular Variables.

We observed an abnormal-inflation effect at the level of singulars, meaning that participants deemed urns *B* and *C*, which contained the lowest proportion of colored balls, more important for bringing about the outcome. Formally, judgments for *B* and *C* were higher than for *A* and *D, t*(1239.25) = −2.56, *p* = 0.010. This qualitative pattern aligned with the predictions of the CESM, but not with the predictions of the NSM, which prescribed abnormal deflation in this context. No significant difference could be observed however between the two low-probability singulars, contrary to the CESM’s expectations (means: 5.31, 5.19; *t*(619.67) = 0.52, *p* > 0.6).

#### The CESM Overestimates the Attractiveness of Some Plurals.

The CESM mistakenly predicted that *A* ∧ *C* should be rated higher than *A* ∧ *B*, and *A* ∧ *B* ∧ *D* higher that *A* ∧ *B* ∧ *C*. In both cases, the predictions come from a tendency of the model to give a very similar rating to the singular *X* and the pair *X* ∧ *Y* if *Y* is a high-probability variable. This is because if *P*(*Y*) is high, the correlation between *X* ∧ *Y* and the outcome is very similar to the correlation between *X* and the outcome. This property often results in erroneous predictions, not only in this particular round, but also in the triple-positive round below, where plurals containing the variable *D* are overestimated. We come back to this pattern in the Discussion section for this experiment.

##### Triple-Positive Round.

In this round, the player drew a colored ball from urns *A, B* and *D* (as in Figure 5b) and therefore won the game. In such a draw, the win is not overdetermined like it was in the previous round, but clearly it is caused by the player’s getting a colored ball from both *purple* urns *A* and *B*. Urn *D*, on the other hand, is not an active cause of the win in the present world, because it has no effect on winning in the absence of *C*.

Notice that, in the particular context of this round, drawing a colored ball from urn *D* does not simply have a low impact on the win, but in a categorical sense it is not at all a cause of the outcome in the actual world. A standard view of how causal-selection judgments work holds that only the events that can be counted as *actual causes* (Halpern, 2016) of the outcome qualify as candidates for causal selection in the first place (see for example Gerstenberg et al., 2021; Quillien & Lucas, 2024). Following this logic, the causal impact score of the event “drawing a colored ball from urn *D*” should simply be zero, and it is unclear if plural events that contain *D* (such as “drawing colored balls from urns *A* and *D*”) should count as actual causes or not. For simplicity, we gloss over this issue, allowing the model to give non-zero causal responsibility to *D* or plurals that feature *D*.

**Figure 7.**
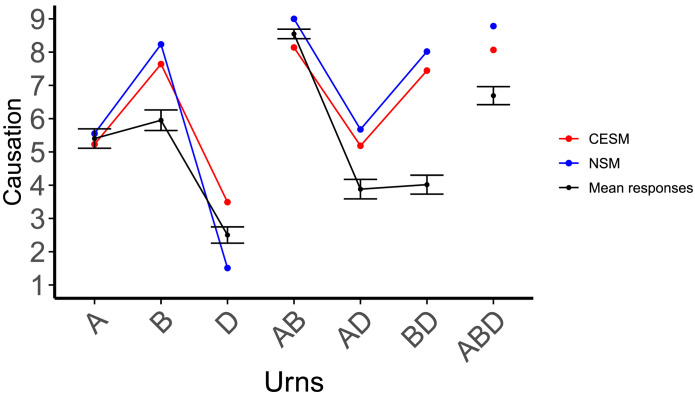
Participants’ responses, along with model predictions, for the triple-positive round. The red line represents the CESM predictions, the blue line the NSM predictions, and the black line represents the mean of participants’ responses.

Figure 7 summarizes the results for the triple-positive rounds. Both counterfactual models give a good account of participants’ judgments: model predictions are correlated with average human judgments *r*(5) = 0.78 (CESM) and *r*(5) = 0.79 (NSM). We now highlight the most significant patterns.

#### Abnormal Inflation Effect for Singulars.

We did observe an abnormal inflation effect, with the low-probability urn *B* being ranked significantly higher than high-probability urn *A* (*t*(617.88) = −2.54, *p* = 0.011), in line with the predictions of both the CESM and the NSM.

#### Ceiling-High Ratings for the Pair *A* ∧ *B*.

Participants were almost unanimous in giving ceiling-high ratings to *A* ∧ *B*. Only 51 participants (out of 311) in total gave it ratings different from the maximal value of the Likert scale.

#### Low Ratings for *D*, and Plurals Containing *D*.

Ratings for the idle variable *D* were very low. More than half of participants (171 out of 311) gave it maximally low ratings. Interestingly however, the ratings weren’t as low as they were high for *A* ∧ *B*, suggesting that the fact that *D* does make a contribution to the win in other possible configurations still had some residual influence on participants’ ratings.

Plurals containing *D*, such as the mixed pairs *A* ∧ *D, B* ∧ *D*, and the triple *A* ∧ *B* ∧ *D*, were systematically rated somewhere between the best cause that they contained and the low ratings of *D*. They were systematically rated lower than predicted by the models, which didn’t penalize strongly enough the inclusion of the idle variable *D*. However, participants didn’t seem to systematically disqualify a plural just for including the variable *D* (for example, by giving it ratings as low as those of *D* alone).

#### Losing Rounds.

The first two conditions just discussed collected judgments about the contribution of *colored ball* draws to a player’s *win* in a given round of the game. The two conditions we present next instead queried participants’ judgments on the contribution of *white ball* draws to a player’s *loss*.

Earlier we discussed the hypothesis that when participants make judgments about a loss, they might simulate counterfactual possibilities in a slightly different way than when they judge a win. In order to formalize this hypothesis in a counterfactual framework, we consider a variant of our computational models featuring a parameter *w*, which encodes participants’ propensity to represent the losing conditions in the non-classical, language-like way depicted in Equation 2, reproduced below.

$${\text{LOSS}}_{\text{strong}}≔\neg A\wedge \neg B\wedge \neg C\wedge \neg D$$

By contrast, the equation for losing obtained by applying the classical negation of the equation for winning is as follows.

$$\begin{array}{l} \text{LOSS}≔\neg \left(\left(A\wedge B\right)\vee \left(C\wedge D\right)\right)\\ \;\equiv \neg \left(A\wedge B\right)\wedge \neg \left(C\wedge D\right) \end{array}$$ (3)

Concretely, we assume that when the outcome under consideration is a loss, the participant makes a random decision in each counterfactual world, where:

- with probability *w*, the loss is determined non-classically (Equation 2);
- otherwise, with probability 1 − *w*, the loss is determined by the classical negation of the original rule (Equation 3). This entails that our earlier *w*-less models can be understood as a special case of the *w* models where *w* = 0;
- once it has been determined whether a given world is an instance of a win or a loss, the worlds that are not losses are recorded as wins. The impact of each variable on the models is then computed exactly as before.

We fitted the models again in this new version using data from all four conditions, via a three-dimensional grid search (*s, w, γ*). The best fitting values were respectively *s* = 0.21 and *w* = 0.77 (with *γ* = 0.41) for the CESM, and *s* = 0.5 and *w* = 0.77 (with *γ* = 1.17) for the NSM. For simplicity, all model predictions we report use the values of the *s* and *γ* parameter fitted jointly with *w*, even for the base versions. Using the original fitted parameters for the base versions yields virtually identical results.

Adding the *w* parameter significantly improved the fit of both models, even accounting for differences in degrees of freedom (Table 6). For the negative conditions below, we report both versions of the models, to showcase the impact of the new parameter.

**Table 6.** Model comparisons for Experiment 2, across all conditions. The *Cor.* column indicates the item-level correlation between model predictions and mean participant responses per question.

Model	LogLik	*χ* ^2^	*p*-value	BIC	Cor.
Baseline	−27494			54997.32	0
CESM, no *w*	−27963	2023.44	<0.0001 ***	55889.97	0.2609
CESM, with *w*	−26905	1175.91	<0.0001 ***	53840.57	0.67
NSM, no *w*	−28390	11511.80	<0.0001 ***	56967.07	0.02
NSM, with *w*	−27634	642.55	<0.0001 ***	55295.31	0.57

#### Overdetermined Negative Condition.

The overdetermined negative condition is the mirror image of the overdetermined positive condition. Here, the player drew a white ball from all four urns, and consequently lost, as pictured in Figure 5c.

**Figure 8.**
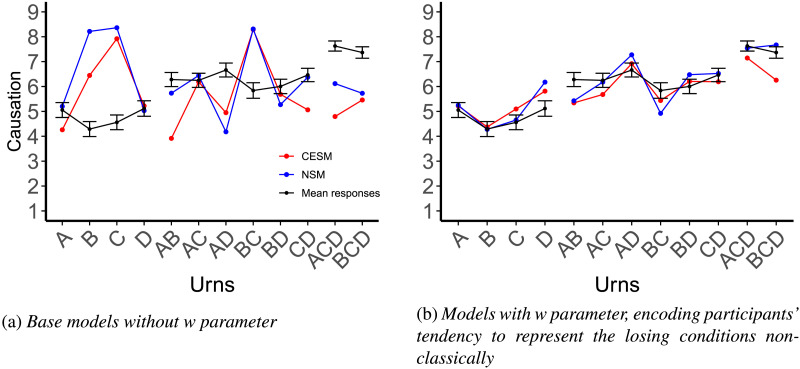
Participants’ responses and model predictions for the overdetermined negative round. The red line represents the CESM predictions, the blue line the NSM predictions, and the black line represents the mean of participants’ responses.

The results are summarized in Figures 8a and 8b. The base versions of the CESM and NSM have a poor fit to participants’ average judgments, *r*(10) = −0.38 (CESM) and *r*(10) = −0.42 (NSM). In contrast, the versions of the models featuring the *w* parameter provide a good account of the data, *r*(10) = 0.82 (CESM) and *r*(10) = 0.87 (NSM); see also Table 7. We now go over our most telling findings.

**Table 7.** Model fits per condition. The *Cor.* column indicates the item-level correlation between model predictions and mean participant responses per question.

Condition	Model	BIC	AIC	Cor.
overdetermined positive	CESM	15009.14	14991.01	0.45
	NSM	15278.45	15260.32	−0.18
triple-positive	CESM	10490.38	10473.32	0.78
	NSM	10505.71	10488.66	0.79
overdetermined negative	CESM, no *w*	19238.14	19225.69	−0.38
	CESM, *w*	17731.07	17718.62	0.82
	NSM, no *w*	19164.41	19151.96	−0.42
	NSM, *w*	17703.51	17684.84	0.87
triple-negative	CESM, no *w*	10853.57	10842.19	0.34
	CESM, *w*	10335.21	10318.16	0.94
	NSM, no *w*	10655.7	10644.33	0.52
	NSM, *w*	10422.88	10405.82	0.99

#### Urns With the Lowest Number of White Balls Are Given Higher Scores.

This effect can be observed both for singulars and for plural causes, with combinations featuring urns *A* or *D* scoring higher than those featuring *B* or *C*. This pattern runs completely contrary to the predictions of counterfactual models under the classical representation of losing conditions from Equation 3, but is captured by the version that assumes a non-classical representation of the losing conditions.

Indeed, if participants are representing losing conditions as a disjunction of minimally sufficient conditions (as in Equation 3), we would expect their judgments to follow the logic of abnormal *deflation* and ascribe a greater causal impact to those urns out of which one is most likely to get a white ball, that is urns *B* and *C*. Instead, their judgments seem to follow a logic of abnormal *inflation*, with a preference for the urns that contain the lowest number of white balls, i.e., *A, D*, consistent with a representation of the losing conditions as a conjunction of necessary events as in Equation 2.

#### No Significant Difference Between Pairs That Cross the Disjunction and Those That Do Not.

Participants’ judgments for pairs that crossed the disjunction (e.g., *A* and *C*) were not significantly different than for pairs that did not cross the disjunction (mean crossing: 6.19; mean noncrossing: 6.37; *t*(1311.86) = −1.47, *p* = 0.140).

This finding is consistent with the idea that participants represent the losing conditions as LOSS*_strong_* ≔ ¬*A* ∧ ¬*B* ∧ ¬*C* ∧ ¬*D*, with no natural grouping of the variables. In contrast, a classical representation of the losing conditions would have predicted that any pair of events on the same side of the purple vs. yellow divide should be redundant, since a single white ball on either side is sufficient to cancel any contribution that this side could have made to a win. There is no such redundancy however if the representation is non-classical, where each white-ball drawing event makes a crucial contribution to the outcome.

#### Triples Are Rated Higher Than Pairs.

Mean pairs: 6.25; Mean triples: 7.37; *t*(1400.78) = −12.64, *p* < 0.0001. Here again, while triples would have been redundant under a classical representation, each element of the triple makes a non-zero contribution to the outcome if the representation is non-classical.

##### Triple-Negative Condition.

In the triple-negative round, the player drew white balls from every urn except for urn *C*, as in Figure 5d. This makes it a mirror image of the triple-positive round, where white balls are substituted for colored balls. In this round, the white ball from urn *D* is indispensable for the loss, whereas urns *A* and *B* are redundant with one another.

The same contrast between classical and non-classical representations of losing conditions applies in this round. Here, the *w* parameter that we enriched our models with encodes participants’ propensity to represent the rule as follows.

LOSS≔¬A∧¬B∧¬D

We take it that the non-classical representation of the losing conditions in this round is slightly different from the overdetermined negative round because, in the actual world, a colored ball *was* drawn from urn *C*. This makes the negation of the plural entity *C* ∧ *D* in our rule harder to represent as the strong plural negation ¬*C* ∧ ¬*D*, since the situation at hand is known to be one where the player in fact drew a colored ball from urn *C*. In other words, the player cannot possibly have lost *because* they drew a *white* ball from *C*, since they in fact drew a *colored* ball from *C*. Our hypothesis then is that participants might negate the rule in a way as homogeneous as possible, without contradicting the established facts about the event.

**Figure 9.**
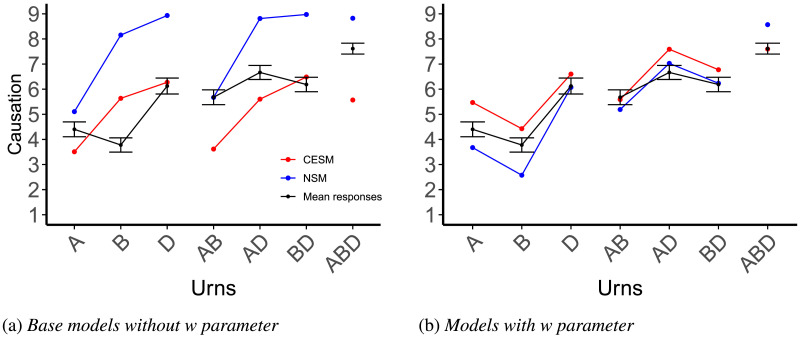
Participants’ responses and model predictions for the triple-negative round. The red line represents the CESM predictions, the blue line the NSM predictions, and the black line represents the mean of participants’ responses.

Results are summarized in Figures 9a and 9b, and in Table 7. The base versions of the CESM and NSM have a moderate fit to participants’ average judgments, *r*(10) = 0.34 (CESM) and *r*(10) = 0.52 (NSM). On the other hand, the versions of the models featuring the *w* parameter provide a good account of the data, *r*(10) = 0.94 (CESM) and *r*(10) = 0.99 (NSM); see also Table 7. We highlight some of the most important qualitative patterns below.

#### Participants Prefer Urns With a Lower Number of White Balls.

Causal judgments for ¬*A* were higher than ¬*B* (*t*(618.98) = 2.98, *p* = 0.003), and causal judgments for ¬*A* ∧ ¬*D* were higher than ¬*B* ∧ ¬*D* (*t*(619.49) = 2.34, *p* = 0.020). The preference for urns featuring a lower number of white balls is similar to what we find in the overdetermined negative round. Again this pattern is most coherent with a non-classical representation of the losing conditions.

#### The Pair ¬*A* ∧ ¬*B* Rates Higher Than Either of Its Constitutive Singulars, and the Triple ¬*A* ∧ ¬*B* ∧ ¬*D* Rates Higher Than Its Constitutive Pairs (*t*(708.60) = 10.27, *p* < 0.0001).

Both patterns are examples of plurals whose effect in the outcome under the classical representation is redundant with that of one of the events (singular or plural) contained within it, which should lead them to be rated at most as high as the sufficient event in question. The fact that these are rated higher by participants is again suggestive of their representing the losing conditions non-classically.

### Overall Model Comparison

Table 6 summarizes the comparison between the models at the global level (all conditions combined). The version of the CESM that includes the *w* parameter has the best fit overall (BIC = 53840.57; correlation with means: *r*(35) = 0.67, *p* < 0.001). This is better than the fit of the model without the *w* parameter (BIC = 55889.97; correlation with means: *r*(35) = 0.26, *p* < 0.001), or than any of the versions of the NSM model (with *w*: BIC = 55295.31, cor.: *r*(35) = 0.57, *p* < 0.001; without *w*: BIC = 56967.07, cor.: *r*(35) = 0.02). In general, the versions of the models that include the *w* parameter are better than the versions without it, by all metrics.

We also compared these models with a constant baseline model, which always made the same predictions for every question in every condition. The prediction was fitted to the data via the scaling parameter *γ* only. All counterfactual models had a better fit than the baseline model when assessed in terms of their correlations with mean human judgments, but only the version of the CESM that included the *w* parameter had a better BIC score than the baseline model.

### Discussion

This second experiment provides more evidence in favor of the psychological reality of plural causes in the context of causal-selection judgments.

Just like in the first experiment, participants’ judgments for plural causes across all four rounds of the game were clearly sensitive to the probabilities attached to the corresponding events. Participants’ judgments in the overdetermined positive round corroborate the non-linearity between participants’ judgments for plurals and their judgments for the singular causes that constitute them. Given the pattern of abnormal inflation observed for singular variables, favoring *B* and *C* over *A* and *D*, a *linear* reconstruction of participants’ judgments for plurals would have us expect the pair *B* ∧ *C* to rank above all others pairs, when in fact it ranks much lower than the *A* ∧ *B* and *C* ∧ *D* pairs.

The winning rounds of the experiment also demonstrate that plural causes featuring more variables are *not necessarily* rated higher than proper subsets of the variables they contain. The Triple-positive round shows a clear pattern in this regard: every time a plural features the variable *D*, its rating is systematically lower than that of the same cause (singular or plural), minus the variable *D*. This contradicts the hypothesis that adding more variables always makes an explanation more attractive, which the results from Experiment 1 could not rule out. And the phenomenon is not limited to the situation where an idle variable like *D* features in a plural: a similar observation can be made about the triplets *A* ∧ *B* ∧ *C* and *A* ∧ *B* ∧ *D* in the overdetermined positive condition, both of which are rated lower than the best pair that they contain, *A* ∧ *B*. Thus, although plurals featuring more variables might be descriptively more thorough, they can still be unappealing if their overall counterfactual dependence profile drops as a result of the variables added.

We also uncovered properties of plural causal judgments that go beyond what is expected based purely on patterns of counterfactual dependence. First, in the winning rounds of our second experiment, participants dislike causal explanations that “cross” the disjunction (*A* ∧ *B*) ∨ (*C* ∧ *D*), above and beyond what is predicted by counterfactual models. The fact that the causal rule features two clearly distinct sufficient conditions seems to exert an influence on participants’ explanatory preferences not fully captured by the counterfactual dependence profile of the variables in question.

Second, the following property of the CESM was not reflected in participants’ judgments. The model tends to give a very similar rating to a singular *X* and the pair *X* ∧ *Y* if *Y* is a high-probability variable. This is because if *P*(*Y*) is high, the correlation between *X* ∧ *Y* and the outcome is very similar to the correlation between *X* and the outcome. This property often results in erroneous predictions, like in the overdetermined positive round where the model predicts (against participants’ judgments) that the pair *A* ∧ *C* should rate higher than the pair *B* ∧ *C*.

Finally, we found that counterfactual models could only account for participants’ judgments in the losing rounds if we hypothesized that participants handle the losing conditions, in their internal computations, in a way inconsistent with the classical-logical negation of the winning conditions. Specifically, participants seem to be representing the negation of the winning conditions (i.e., the losing conditions) in a way consonant with how natural-language represents the negations of plurals.

## GENERAL DISCUSSION

Humans make systematic judgments regarding which of several events influencing an outcome should be considered as *the cause*, or the most important cause of that outcome. These *causal selection* judgments are the object of a rich and actively expanding section of the psychological literature on actual causation. So far, however, this literature has been exclusively focused on *singular* events, identified with the distinct nodes of the relevant causal system. In this article, we argue that its scope should be extended to include *plural* events, featuring multiple variables.

Our experiments present strong evidence that judgments about plural events cannot be captured in terms of linear combinations of the judgments for the events that constitute them. There appears to be no obvious way of combining participants’ causal judgments regarding any two events *A* and *B* that would predict their judgment for the event “*A and B*.” Our results thus establish the psychological reality of plural causes: plural causes are treated by the mind as causal entities in their own right, and their impact on the outcome is apprehended in a *holistic* or wholesale fashion. The lesson here is that people’s assessment (and likely also their production) of causal explanations is not constrained by the boundaries of individual variables but instead can operate at the level of multivariate groupings.

A second set of observations looks at the ways in which such groupings differ from classical conjunctions of variables. In positive outcomes, groups of variables that cross over sufficient conditions for producing an outcome seem to be disfavored. Even more striking are judgments in negative outcomes, where the default expectations of counterfactual models are reversed, unless one assumes that subjects assess causes by their contribution to a representation of losses that is altogether baffling under standard classical-logical expectations, but understandable if plural causes function like plurals in natural language.

### Summary of Our Findings and Their Immediate Consequences

#### Plural Cause Judgments Cannot Be Reconstructed as Linear Combinations of Singular Judgments.

It seems *prima facie* plausible that, when people make a causal judgment about whether “*A* and *B* caused *E*,” they might judge how much *A* caused *E*, judge how much *B* caused *E*, and then combine these two judgments into a single judgment for the plurality. Under this view, plural causal selection would be entirely predictable from facts about singular causal selection. One of our main goals was to rule out this null hypothesis.

In our two experiments we designed situations in which computational models predict that, if plurals are processed in a holistic manner, judgments about plural causes should not be simple combinations of judgments about their constituent singular variables. Participants’ judgments in these situations supported this prediction, successfully ruling out the linear-combination hypothesis.

#### Counterfactual Models Can Account for a Broad Range of Plural Causation Judgments.

A growing body of research provides strong evidence that causal judgment involves counterfactual thinking (e.g., Gerstenberg et al., 2017; Kahneman & Miller, 1986; Krasich et al., 2024; Quillien & Lucas, 2024). At the same time, there are debates about what phenomena counterfactual theories can explain (Hall, 2004; Henne, 2024; Lombrozo, 2010; Rose et al., 2021; Sytsma, 2020), and about the computations that counterfactuals might be an input to (Icard et al., 2017; Quillien, 2020).

Our experiments provide a rich opportunity to probe the scope and the generality of counterfactual theories. None of the counterfactual theories that we are aware of were developed with the goal of explaining data about how people make plural causation judgments. Consequently, accounting for these judgments off-the-shelf would constitute important evidence in favor of these theories.

We found that two recent counterfactual models of causal judgment (Icard et al., 2017 and in particular Quillien & Lucas, 2024) can quantitatively account for many features of participants’ judgments. In particular, when participants’ judgments about plurals diverge from a linear combination of their constituent singular causes, they typically do so in the way that is predicted by the counterfactual models. As such, our results strengthen the case for counterfactual theories.

The losing rounds of our second experiment did however offer a more ambivalent verdict. On the one hand, counterfactual models used off the shelf performed very poorly and even predicted patterns of preferences that were the exact opposite of those observed. On the other hand, the same models could be made to track those patterns very well when complemented with a specific hypothesis about how people score causes in negative outcomes, more on which shortly. The plausibility of a counterfactual account of these data thus depends on the plausibility of this additional assumption.

#### Causal Judgments Favor Sets of Variables Belonging to the Same Disjunct.

In the game that participants played in Experiment 2, the player needed to draw either two purple or two yellow balls in order to win the game. From a logical point of view, this rule is a *disjunction*: the player wins if either one of the conditions for victory is met; each condition is a *disjunct*.

Participants favored plural causes that do not cross the boundary between the two disjuncts. Suppose for example that the player drew purple balls from urns *A* and *B* and yellow balls from urns *C* and *D*. In this situation, participants would be reluctant to say that the player “won because he drew a colored ball from urn *B* and urn *C*.” The counterfactual models also disfavored these boundary-crossing plurals, but participants did so to an even greater extent than predicted.

There are different possible explanations for this pattern. At a superficial level, for example, participants might have preferred causal explanations that mentioned balls of the same color because of low-level perceptual biases.

**Figure 10.**
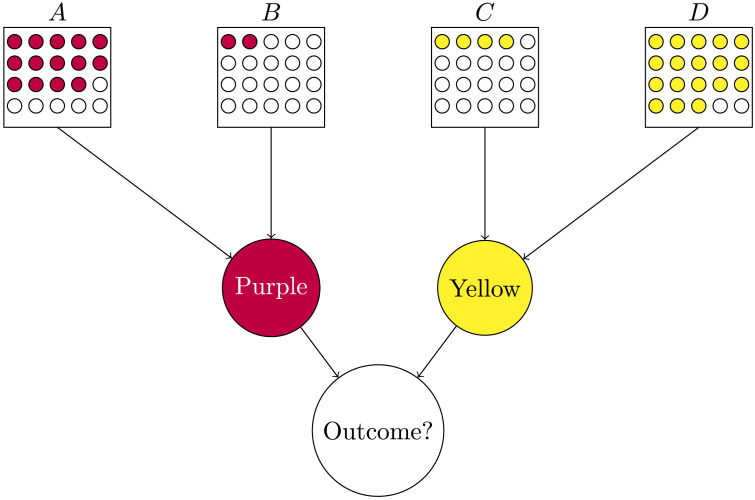
Causal graph representing participants’ putative model of the situation.

At a deeper level, participants might have built an internal representation of the game in terms of a causal model with a particular structure. This causal model would contain intermediate variables (in the technical, causal-model sense of “variable,” Pearl, 2000; Woodward, 2016) representing whether each condition for victory (getting two purple balls and getting two yellow balls) is met, see Figure 10. Such a model would be distinct from one without the intermediate variables, in that it would support new kinds of interventions unavailable to the more straightforward model. For example, one could in principle intervene to set the variable corresponding to “Both yellow balls,” i.e., *A* ∧ *B*, to TRUE even though one yellow ball is not present. But this seems like a paradoxical causal model, as it features variables that hold a containment relation to one another (see e.g., Beckers et al., 2023, for how this can introduce problems for structural models in general).

An alternative, related hypothesis is that people represent the groupings *A* ∧ *B, C* ∧ *D* not as distinct intermediate causal variables, but as distinct processing *pathways* for deriving outcomes from variables *A, B, C, D* in accordance to the causal model WIN ≔ (*A* ∧ *B*) ∨ (*C* ∧ *D*). This hypothesis is in line with accounts in the mental model theory tradition (see in particular Khemlani et al., 2018) which propose that people represent disjunctions by representing each disjunct within a mental model. For example, to represent the disjunction (*A* ∧ *B*) ∨ (*C* ∧ *D*), people would picture two distinct mental models, *A* ∧ *B* and *C* ∧ *D*, and assess whether the disjunction is true by checking whether the conditions specified by one of the model are satisfied.

There is independent evidence that this structured way of representing disjuncts can account for several surprising patterns of reasoning with disjunctions (Chung et al., 2022; Koralus & Mascarenhas, 2013; Walsh & Johnson-Laird, 2004). Studies of deductive reasoning have investigated how people reason about logical statements of the shape (*A* ∧ *B*) ∨ *C*. In experiments replicated and varied multiple times, participants overwhelmingly conclude *B* from the two premises (*A* ∧ *B*) ∨ *C*, and *A* (Koralus & Mascarenhas, 2018; Picat & Mascarenhas, 2024; Sablé-Meyer & Mascarenhas, 2021; Walsh & Johnson-Laird, 2004). But this is a fallacy: it is compatible with the premises but not the conclusion that *A* and *C* should be true while *B* is false. Koralus and Mascarenhas (2013) explain this fact in terms of question-answer dynamics: the disjunction in the first premise is naturally interpreted as demanding the participant *choose* between one of the two disjuncts. This in turn induces dependencies between propositions occurring *within disjuncts*: in the context of (*A* ∧ *B*) ∨ *C*, the second premise *A* is seen as an answer in the *A* ∧ *B* direction, introducing dependence between *A* and *B*. In general, this approach predicts that the conjuncts within each disjunct will be taken, as it were, to *hang together* in a cohesive way, so that learning about one will constitute evidence in favor of all of the others.

There is even evidence of such effects absent the language of disjunction, in experiments where the same information was conveyed by means of visual stimuli in the form of animations (Chung et al., 2022), indicating that this “packaged” way of representing a disjunction is not simply a fact about the interpretation of the word “or” and its equivalent locutions. Rather, these rich, structured disjunctive representations which induce dependencies not predicted by standard Boolean interpretations of logical connectives are available to human minds far more generally. In particular, they will have been available to participants in our Experiment 2, and may have played a part in shaping their causal judgments, by pushing them to associate *A* and *B* on the one hand and *C* and *D* on the other more tightly than is predicted by classical accounts of disjunction, whether deductive or probabilistic.

#### Plural Causes Featuring More Events Are Not Necessarily Better.

In Experiment 1, we found that participants preferred causal explanations that mentioned the most causes, and that this preference was stronger than predicted by counterfactual models. We probed the extent of this trend in Experiment 2, where we found that mentioning more causes does not always make a causal explanation better. For example, a causal explanation mentioning only two events *A* and *B* might be judged better than an explanation mentioning *A, B*, and *C*.

These results suggest that causal judgments are subject to a trade-off between two different considerations (see also Kirfel et al., 2024; Sumers et al., 2024). On the one hand, people might favor explanations that give detailed information about what events happened. Since every explanation of the shape “*X* happened because *Y*” comes with the implication that “*Y* happened” (Halpern, 2016), causal explanations that feature many causes offer more complete descriptions of what happened. With respect to this criterion, plural causal explanations are always more helpful than singular ones, since they highlight more true facts about the situation.

On the other hand, causal explanations convey information about patterns of counterfactual dependence (Quillien, 2020). Under this criterion, large plural causes can sometimes be worse. For example, an explanation mentioning three events *A, B*, and *C* might misleadingly suggest that the outcome strongly covaries with the conjunction of these three events, across counterfactuals.

#### Judgments for the Losing Rounds Suggest a Non-Standard Representation of Losing Conditions.

In the trials of Experiment 2 where the player loses the game, we could not account for the data by assuming that participants internally represent the conditions for losing the game (for the purpose of simulating counterfactual possibilities) as the classical complement of the conditions for winning. Instead, judgments can be captured quite adequately if we suppose that, in the losing rounds, participants score various causes to the extent that they contribute to a different target, which is a situation in which the player draws a white ball from all urns.

This target does not correspond to the classical-logic negation of the winning conditions and does not track with what people know to be the actual minimal conditions for losing a round of the game. But it is consistent with the assumption that, to assess causes of negative outcomes, people track how various causes match with the *linguistic plural* negation of the disjunction or set of models {*A* ∧ *B*, *C* ∧ *D*} they would use to assess their contribution to positive outcomes. This suggests that the representation participants use for deriving an outcome might possess a semantics similar to that of natural-language plurals, in that these conjunctions are treated as homogeneous entities, and negated as such. Note, in this connection, that the effects we find are unlikely to stem from linguistic experimenter demands when interpreting the causal statements verbally. We asked participants about the causal impact of “drawing a white ball” on a “loss,” never about the impact of “not drawing a colored ball” on “not winning.”

Additional theoretical and experimental work is needed to put this particular hypothesis about plural negation on firmer ground, and our general thesis about the psychological reality of plural causes does not depend on it. For now, we observe that this hypothesis is a first step toward enriching models of causal judgment. It points to the need for more sophisticated considerations about the representational tools people draw on when simulating counterfactuals.

Typically, counterfactual models have assumed that we can gloss over the particular way that humans represent logical connectives (“and,” “or,” and so on) when they simulate counterfactual possibilities, with the implicit premise that these representations jibe well enough with the normative standards from classical logic. This assumption is reasonable: if there is a “language of thought” (Fodor, 1975; Quilty-Dunn et al., 2023), one would expect it to operate more like a programming language or formal logic than like natural language, especially for purely internal operations where no communication is involved.

What makes our results surprising, then, is that people’s representations of losing conditions appear to follow patterns more reminiscent of natural-language plural semantics than of classical logic. To be clear, we are not *assuming* that the language of thought resembles natural language. We are observing, first, that everyone assumes that it does *not*, and second that, at least in this domain, it seems to. Whether the mental representations involved in causal judgment have a language-like format more broadly is a deeper question that we do not attempt to settle here. But this observation invites a broader methodological point: work on the formal semantics of natural language may be a more fruitful source of hypotheses about psychological phenomena than is typically assumed (Goodale & Mascarenhas, 2026; Wellwood & Hunter, 2023).

## CONCLUSION

The current study is a preliminary demonstration that, to the mind, causes are more than the sum of their parts. Judgments about plural causes are affected by the prior probability of their constituent variables, but cannot be derived from the causal strength of these individual variables. Our results are consistent with simple extensions of extant counterfactual models of causal selection. At the same time, our findings raise new issues about the psychology of causation, which point to opportunities for extending existing theories. We anticipate that future research on the psychology of plural causation will open yet further avenues of inquiry about human causal cognition.

## ACKNOWLEDGMENTS

We are very grateful to Jonathan Kominsky for extremely helpful, detailed comments on several iterations of this manuscript, as well as to several other anonymous reviewers whose feedback greatly improved the paper. We also wish to thank Thomas Icard, Michael Goodale, and the audience of the LANG-REASON seminar at Institut Nicod for discussion that greatly contributed to the development of this work.

## FUNDING INFORMATION

This work was supported by Agence Nationale de la Recherche grants ANR-18-CE28-0008 LANG-REASON (PI: Mascarenhas) and ANR-19-P3IA-0001 PRAIRIE 3IA Institute (PI: Mascarenhas).

## AUTHOR CONTRIBUTIONS

C.K.: Conceptualization; Data curation; Formal analysis; Investigation; Methodology; Visualization; Writing – original draft; Writing – review & editing. T.Q.: Conceptualization; Formal analysis; Methodology; Writing – original draft; Writing – review & editing. S.M.: Conceptualization; Data curation; Funding acquisition; Methodology; Writing – original draft; Writing – review & editing.

## DATA AVAILABILITY STATEMENT

All data and experimental materials for the studies in this manuscript are available on the Open Science Framework at https://osf.io/43m5d/.

## Notes

^1^ Our Experiment 1 was presented at the Forty-fifth Annual Meeting of the Cognitive Science Society and published in the society’s non-archival proceedings (Konuk et al., 2023).^2^ This study was reported at the Forty-fifth Annual Meeting of the Cognitive Science Society and published in the society’s non-archival conference proceedings (Konuk et al., 2023). Our writing in this section borrows directly from this preliminary report.
